# “Dream Insertion”: A Case Report of a Novel Psychotic Symptom in a Male Inpatient With Treatment Resistant Schizophrenia

**DOI:** 10.1155/crps/3048932

**Published:** 2026-01-16

**Authors:** David Clayton, Sandeep Grover

**Affiliations:** ^1^ Department of Mental Health, Northern Hospital Epping, Melbourne, Victoria, Australia, nh.org.au

**Keywords:** case report, delusion, dream insertion, dreaming, psychosis, schizophrenia, thought insertion

## Abstract

**Background:**

Individuals with schizophrenia appear to experience differences in dreaming compared to control populations—the small amounts of research that does exist demonstrates abnormalities such as simpler dreams that are more bizarre and with greater negative content than those of controls. Dream‐related psychopathology remains insufficiently described however—particularly experiences in which patients believe their dreams are externally generated or controlled. Such phenomena extend classically described first rank symptoms—such as thought insertion and passivity phenomena—into the domain of sleep mentation. This case presents a patient who describes a delusional belief that an external agent can directly infiltrate his nocturnal state to insert dreams themselves.

**Case Presentation:**

A 34‐year‐old male patient with schizophrenia was admitted to the inpatient unit for commencement of clozapine following poor response to multiple antipsychotic agents. While admitted, he described a new delusional belief that a Central Intelligence Agency (CIA) agent was infiltrating his dreams at night and inserting externally created dreams. This symptom did not respond to ongoing clozapine uptitration and community follow‐up suggests an ongoing limited response.

**Conclusions:**

This patient describes a novel symptom not previously described in the literature to the best of the authors knowledge and neither appears consistent with other commonly described sleep related phenomenon nor first rank symptoms. As such, we use the term “dream insertion” to describe the phenomenon noted in the case report and suggest an expanded inquiry into the oneiric effects of schizophrenia to enhance understanding and management of such symptoms.

## 1. Introduction

Schizophrenia is a major psychiatric disorder characterized by positive symptoms, negative symptoms, and progressive cognitive impairment [1]. Sleep abnormalities are experienced by the majority of patients and have gained prominence in clinical research [2]. Disrupted sleep is frequently reported during the prodromal phase and may predict acute symptom exacerbations [3–5].

There are several sleep‐related experiences that resemble psychotic phenomena, such as hypnagogic and hypnopompic hallucinations, though these are typically nonpathological and not externally attributed [6]. Nightmares and night terrors similarly occur in nonpathological contexts but appear at elevated rates in psychiatric disorders [7]. Childhood nightmares have also been associated with later development of psychosis [8]. Alongside this, experimental early research also demonstrated that severe sleep deprivation can produce symptoms of psychosis in healthy individuals in the form of hallucinations, paranoia, and cognitive disorganization [9].

Studies indicate that patients with schizophrenia may experience shorter dream reports, impoverished content, and greater bizarreness compared with controls [10]. However, understanding psychotic‐like phenomena within dreams requires careful differentiation. Dream content in healthy individuals often includes implausible or bizarre elements, meaning that unusual dream narratives are not inherently pathological. What becomes clinically relevant however is the waking interpretation—particularly when an individual attributes creation or agency of dreams to an external source. This distinction is essential in determining whether a phenomenon is best understood as nonpathological; a parasomnia or a novel psychotic symptom.

In this case report, we present a patient with the delusional belief that an external agent can directly infiltrate dream states to insert dreams themselves. To our knowledge, this manifestation has not previously been described in the literature.

## 2. Case Description

### 2.1. Background

A 34‐year‐old male of Yemeni heritage was admitted to a public inpatient psychiatric unit for initiation of clozapine after multiple failed adequate antipsychotic trials. He was diagnosed with treatment‐resistant schizophrenia and had a total of eight previous inpatient admissions prior to his current presentation. His illness was characterized by delusions of persecution from a “man in the shadows”; auditory hallucinations of a male voice; negative symptoms; chronic poor insight and marked psychosocial dysfunction.

His childhood history revealed physical abuse by parental figures as well as displacement from his native Yemen due to conflict. Despite this exposure to childhood trauma, he did not endorse symptoms on screening tools for post‐traumatic stress disorder. There was no history of other psychotic symptoms; affective symptoms; obsessive compulsive symptoms nor sleep‐related disorders. Alongside this, his physical examination was noted to be unremarkable.

### 2.2. Mental Status Examination

On initial review, he disclosed a previously undocumented belief that a “Central Intelligence Agency ([CIA] agent)” was targeting him via his dream content. According to him “it was like a revelation when I was dreaming. I noticed his face in a crowd in the dream and I went to speak to him directly where he revealed he was a CIA agent.” He was concrete in his belief that this “CIA agent” had been operating in this manner the entirety of his illness. According to him, the agent did not resemble anyone he knew or whom he had seen in reality. He insisted that his dream content was now being manipulated by this same man—“he uses psychological technology he gained from extensive CIA training to infiltrate my dreams. I’ll have my normal dreams and then suddenly he’ll insert the hallway of his building into mine and I can hear his voice speaking to me. Other times he’ll insert imagery almost like video clips—a man drowning in a river or a woman in a headscarf holding up a seven fingered hand.”

On inquiry, he clarified that the experience would usually occur after few hours of going to sleep; would not lead to waking up from sleep itself; was not associated with any automatisms, vocalizations, nor movements and were experiences that he seemed to have consistently good recollection of when waking. The dream narratives were described as vivid and intrusive; however, the specifics were not clinically central.

He was unable to discern any symbolic significance to any of the dream content to aspects of his life but was unwavering in his view that “these dreams are not mine and they are his work.” He further described the man as he being “an obese American man in his late 50s who was overseeing a team of 10–15 agents who all sleep and work in the same windowless building … the dream factory.”

When inquired how he differentiates this feeling of the CIA agent inserting dreams versus the normal sense of passivity that can be experienced in nonlucid dreaming, he reported that—“I recognize his interference in my dreams because they feel different to my own. It’s like things he puts in my dreams has his own signature that I can feel.” When asked why he is being targeted, he explained that—“he’s doing this to stunt my progress and stop me from achieving my goals in life. He does this because he doesn’t want to see me succeed. My goals in life are being obstructed because I’m exerting so much mental energy battling the CIA agent at night.” At successive reviews, he could not be convinced against any of his beliefs.

### 2.3. Treatment Course

His routine investigations (full blood count, urea and electrolytes, c‐reactive protein, liver function tests, thyroid function tests, fasting blood glucose, and lipid profile) and magnetic resonance imaging of the brain did not reveal any abnormality. He was found to have low vitamin D levels at 22 nmol/L however, which were supplemented accordingly. Polysomnography and electroencephalography were considered; however, the patient declined.

He was commenced on clozapine with the dose gradually increased to 300 mg/day over the next 4 weeks. His serum clozapine levels at this dose were found to be 372 µg/L and norclozapine level was 324 µg/L; however, the dose could not be increased further due to excessive sedation. He did not perceive any improvement in his symptoms; however, he was agreeable to continue taking clozapine following discharge. In successive community follow‐up, no significant changes were noted in his reported symptoms despite 3 months of ongoing clozapine treatment.

## 3. Discussion

This patient presenting with a delusion of an external agent inserting dreams themselves into the nocturnal mind has not been described in the literature to the best of the authors’ knowledge. We considered a number of differentials, however, could not fit the experience into commonly described psychotic nor sleep related phenomena.

We considered the possibility of hypnagogic/hypnopompic hallucinations, which occur at sleep onset or offset, respectively. This patient’s experiences, however, were reported to occur mid‐sleep alongside an association with an external attribution. The description also was not akin to nocturnal temporal lobe epilepsy, which is typically associated with vocalization, automatisms, and confusion—none of which were noted in this patient [11]. Additional consideration was given to lucid dreaming, which is characterized by a high degree of self‐agency rather than external attribution and similarly without delusional interpretation [12].

Dream insertion may represent an extension of first rank passivity phenomena—in which individuals believe actions, thoughts, and sensations are influenced or controlled by an external agent [13]. Whereas thought insertion relates to cognitions during the wakened state; dream insertion involves misattribution of dream content. Agency misattribution models propose that schizophrenia involves a disturbance in self‐monitoring, leading to externally attributed internal experiences [14]. Extending these models to dreaming suggests that internally generated dream mentation may similarly be misattributed. Dreams may naturally incorporate waking fears or beliefs; therefore, psychotic themes within dreams are not unexpected [15]. However, the belief that dreams are externally created and inserted is qualitatively distinct. As such, we use the term ‘dream insertion’ to describe this described phenomenon.

Several studies describe the phenomenological similarities between the dream state and psychosis—noting the shared internally generated visual and sensory phenomena often associated with impaired executive functioning; a decreased locus of self‐control alongside incongruent emotions—postulating that the dream state can be seen as a useful model for psychosis itself [16, 17]. Additionally, some researchers speculate that dream reporting conditions—such as the commonality of working memory failure within dreams—may be a key factor limiting the reporting of oneiric phenomena compared to the wakened state [18]. Alongside this, multiple studies demonstrate a further reduced capacity for dream recall and lesser dream involvement in schizophrenia compared to control populations [10, 19]. This could provide a partial explanation as to why “dream insertion” has not previously been described in the literature.

We do note limitations driven by the single case nature of the report; whereas, a case series would allow for comparing various phenomenological accounts. Utilizing polysomnography or actigraphy would have allowed for objective assessments of the patient’s sleep; however, he was not accepting of these during admission. Alongside this, use of formalized rating scales for psychosis such as the Positive and Negative Syndrome Scale (PANSS) could have allowed for a quantifiable measurement of response in his symptoms—whereas this case report featured a series of clinical assessments. It would also have been pertinent to explore the trauma‐related and cultural influence on interpretation of dream content—noting that narratives involving intelligence agencies may represent themes relevant to experiences with wartime conflict and trauma exposure such as perceived threat, reduced agency, or bodily vulnerability.

Studies fail to demonstrate clear markers that would predict response to clozapine—however a multitude of factors such as pharmacokinetics, genetics, comorbidities, or even metacognitive inflexibility may act as influencers on responsiveness [20]. Given the limited response to clozapine in the case report, exploration of psychological interventions with an evidence basis of utilization for treatment‐resistant psychotic symptoms—such as cognitive behavioural therapy for psychosis (CBTp)—may be considered to assess efficacy in ongoing targeting of dream insertion as a symptom [21]. Future research may benefit from phenomenological studies examining the qualitative features of dream content in psychosis—including the boundaries between internally generated and externally attributed dream experiences. In parallel, neurophysiological studies—using sleep‐stage monitoring, electroencephalography or targeted awakening protocols—could clarify whether abnormalities in REM‐related processes contribute to such phenomena [22]. Additionally, the development and evaluation of therapeutic approaches that explicitly address dream‐related delusional interpretations may provide new avenues for managing similar cases where conventional pharmacotherapy offers limited benefit.

## 4. Conclusions

To conclude, this case demonstrates a novel psychotic symptom characterized by the delusional belief that dreams themselves are being implanted by an external agent. This early phenomenologically driven report suggests “dream insertion” as a symptom distinct from other psychotic or sleep related phenomena and suggests a need for expanded inquiry into the oneiric effects of schizophrenia and the role of pharmacological and psychosocial strategies in managing such symptoms.

## Ethics Statement

Approval from Northern Health’s Research Development and Governance Unit was gained prior to writing and publication of this case report.

## Consent

Patient consent was obtained for publication. Patient anonymity has been maintained, and no personally identifiable information is included in this report.

## Conflicts of Interest

The authors declare no conflicts of interest.

## Author Contributions

**David Clayton**: writing, analysis. **Sandeep Grover**: editing, analysis.

## Funding

No funding was received for this manuscript.

## Data Availability

Data sharing is not applicable to this article as no datasets were generated or analyzed during the current study.
